# Differential Regulation of MicroRNAs in End-Stage Failing Hearts Is Associated with Left Ventricular Assist Device Unloading

**DOI:** 10.1155/2015/592512

**Published:** 2015-02-01

**Authors:** Cristina Barsanti, Maria Giovanna Trivella, Romina D'Aurizio, Mariama El Baroudi, Mario Baumgart, Marco Groth, Raffaele Caruso, Alessandro Verde, Luca Botta, Lorena Cozzi, Letizia Pitto

**Affiliations:** ^1^Institute of Clinical Physiology, National Research Council, CNR, Via Moruzzi 1, 56124 Pisa, Italy; ^2^Institute of Life Sciences, Scuola Superiore Sant'Anna, Piazza Martiri della Libertà 33, 56127 Pisa, Italy; ^3^Laboratory of Integrative Systems Medicine, National Research Council, Via Moruzzi 1, 56124 Pisa, Italy; ^4^Fritz Lipmann Institute for Age Research, Leibniz Institute, Beutenbergstraße 11, 07745 Jena, Germany; ^5^Institute of Clinical Physiology, National Research Council, Cardiothoracic and Vascular Department, Niguarda Ca' Granda Hospital, Piazza Ospedale Maggiore 3, 20162 Milan, Italy; ^6^Cardiothoracic and Vascular Department, Niguarda Ca' Granda Hospital, Piazza Ospedale Maggiore 3, 20162 Milan, Italy

## Abstract

Mechanical unloading by left ventricular assist devices (LVADs) in advanced heart failure (HF), in addition to improving symptoms and end-organ perfusion, is supposed to stimulate cellular and molecular responses which can reverse maladaptive cardiac remodeling. As microRNAs (miRNAs) are key regulators in remodeling processes, a comparative miRNA profiling in transplanted hearts of HF patients with/without LVAD assistance could aid to comprehend underlying molecular mechanisms. Next generation sequencing (NGS) was used to analyze miRNA differential expression in left ventricles of HF patients who underwent heart transplantation directly (*n* = 9) or following a period of LVAD support (*n* = 8). After data validation by quantitative real-time PCR, association with functional clinical parameters was investigated. Bioinformatics' tools were then used for prediction of putative targets of modulated miRNAs and relative pathway enrichment. The analysis revealed 13 upregulated and 10 downregulated miRNAs in failing hearts subjected to LVAD assistance. In particular, the expression level of some of them (miR-338-3p, miR-142-5p and -3p, miR-216a-5p, miR-223-3p, miR-27a-5p, and miR-378g) showed correlation with off-pump cardiac index values. Predicted targets of these miRNAs were involved in focal adhesion/integrin pathway and in actin cytoskeleton regulation. The identified miRNAs might contribute to molecular regulation of reverse remodeling and heart recovery mechanisms.

## 1. Introduction

MicroRNAs are highly conserved, single-stranded, noncoding RNAs of about 18–24 nucleotides, which act as endogenous repressors of target genes, either by inhibiting translation or by promoting degradation of the mRNA [1]. An individual miRNA can influence hundreds of gene transcripts to coordinate complex programs of gene expression and, thereby, effect global changes in the physiology of a cell [2, 3]. Due to their extraordinary variability of expression across tissues and physiological states, miRNAs can be powerful diagnostic and prognostic tools in several diseases. Accordingly, alterations in intra/extracellular miRNAs have been correlated with various cardiovascular conditions, such as myocardial infarction, hypertrophy, cardiomyopathy, and arrhythmias [4].

In advanced HF, mechanical circulatory support by LVADs may bring beneficial effects through unloading of failing left ventricle (LV), increasing total cardiac output, and improving general clinical condition and potentially quality of life. In addition, LVAD support is reported to stimulate cellular, biochemical, and molecular changes in the failing heart, which can result in improvements in different aspects of myocyte functionality, such as calcium handling, responsiveness to beta-adrenergic stimulation, and contractile properties [5, 6]. This complexity of cellular and molecular responses is globally referred to as “reverse remodeling,” and it is considered to be possible mechanism of heart function recovery, which in some cases can lead to LVAD weaning.

Since changes in gene expression and regulation are involved in all remodeling processes, in the last years research has been dedicated to the investigation, at a molecular level, of mechanisms which could be altered during HF progression but could be also susceptible to recovery after mechanical assistance [7–12]. Although miRNA profiling of failing hearts with or without LVAD support has given so far contradicting results, probably because of the different approaches used (Q-RT PCR [7, 13], microarray [8, 11], and high-throughput sequencing [10, 12]) and the variability of patients' conditions, these results support the hypothesis that microRNAs are important players in LVAD-induced heart remodeling and sustain further studies for the continued exploration of this class of gene regulators.

The aim of this study was to evaluate and compare miRNA expression profiling in heart tissues of advanced HF patients undergoing cardiac transplantation directly (HTx-CTRL) or after a period of support with LVAD as bridge to transplant (HTx-LVAD). Next generation sequencing techniques have been applied for a complete qualitative and quantitative analysis of miRNA expression, in order to check possible differentially modulated miRNAs, capable of distinguishing the two groups of patients.

## 2. Materials and Methods 

### 2.1. Study Population and Tissue Sample Collection

The study was retrospectively performed on heart tissues collected at the time of heart transplantation from advanced HF patients who underwent organ transplantation directly (HTx-CTRL, *n* = 9) or after a period of support with a LVAD as bridge to transplant (HTx-LVAD, *n* = 8). Patients were recruited at the Cardiothoracic and Vascular Department of Niguarda Ca' Granda Hospital in Milan, Italy. The study was approved by the local ethics committee. A written informed consent was obtained from each patient. The age of patients ranged from 18 to 65 years; the diagnosis of advanced HF (NYHA class III-IV) was due to idiopathic dilated cardiomyopathy (IDC) or ischemic cardiomyopathy, that is, dilated cardiomyopathy induced by chronic ischemic heart disease with/without history of myocardial infarction (IHD) (Table 1). The patients showed an ejection fraction <35% at the moment of heart transplantation or LVAD implantation. Exclusion criteria were hypertrophic cardiomyopathy, dilated cardiomyopathy secondary to genetic syndromes, fixed pulmonary hypertension, irreversible renal or hepatic failure, severe diabetes mellitus with end-organ damage, severe peripheral vascular or cerebrovascular disease, and coexisting active neoplasm.

Hemodynamic (from right heart catheterization) and echocardiographic parameters were derived from the clinical database and acquired at the time of patient insertion in the transplant list. Moreover, in the LVAD implanted group, data were obtained twice, at LVAD implant and off-pump (clamping outflow cannula) at the time of heart transplant, immediately before native heart removal. Pretransplant measurements derived from transthoracic echocardiography were acquired during LVAD hemodynamic unloading. Seven patients were supported by continuous flow LVADs: 4 by HeartMate II LVADs (Thoratec, Pleasanton, CA), 2 by De Bakey LVADs (MicroMed Technology, Houston, TX), and 1 by INCOR LVAD (Berlin Heart AG, Germany); one patient received Best BEAT (NewCorTec, Pomezia, Italy) pulsatile LVAD pump. All patients in the study were on optimal pharmacological treatment.

In both groups of patients, tissue samples from LV anterior or lateral basal zone were taken at the moment of heart transplantation, immediately frozen in liquid nitrogen, and stored at −80°C.

### 2.2. RNA Extraction and miRNA Sequencing

Heart tissue samples ranging from 30 to 120 mg were used for RNA extraction. Tissues were powdered and resuspended in QIAzol Lysis Reagent (Qiagen). Total RNA extraction was performed by phenol-chloroform extraction methods using miRNeasy kit (Qiagen), according to the manufacturer's instructions. Quality control of total RNAs was performed with the RNA 6000 pico kit (Agilent Technologies 2100 Bioanalyzer). Small RNA libraries were constructed with the TruSeq Small RNA kit (Illumina), according to the manufacturer's instructions. Sequencing was performed using Illumina's HiSeq 2000.

### 2.3. MiRNA Profiling and Differential Expression Analysis

Raw sequences of 50 bp length were produced and demultiplexed using the Illumina pipeline CASAVA v1.8. FastQC v0.10.1 (http://www.bioinformatics.babraham.ac.uk/projects/fastqc/) was used for quality check. Primary reads were initially trimmed off adapter sequences using Cutadapt v.1.2.1 [14]. Remaining high quality reads, with length of 17–35 bp after clipping, were clustered for unique hits and mapped to known human pre-miRNA sequences from mirBase (release 20) employing miRExpress tool (v2.1.3) [15]. The 95% of sequence identity between reads and pre-miRNA sequences and length tolerance shifting range of 4 bp were considered acceptable. miRNA expression profiles were built by calculating the sum of read counts for each miRNA, according to the alignment criteria.

To inspect sample relations, variance-stabilizing transformed count data were used to build an Euclidean distance matrix, followed by hierarchical clustering analysis, as described elsewhere [16]. miRNA differential expression analysis was performed using Bioconductor's package DESeq [16]. The obtained read counts for each identified miRNA were first normalized by scaling for library size factors in order to deal with variation among samples. An independent filter was then used to improve the detection power of the statistical test [17], as suggested in the DESeq vignette [18]. Specifically, the overall sum of counts, irrespective of biological condition, was considered in order to remove miRNAs in the lowest 30% quantile. *P* values were estimated using a negative binomial distribution model and local regression to estimate the relationship between the dispersion and the mean of each miRNA. Raw *P* values, considered as statistically significant when <0.01, were finally adjusted for multiple testing by the Benjamini and Hochberg procedure [19], by setting a 10% false discovery rate (FDR) threshold. A fold change (FC) threshold of 1.6 was finally used to select modulated miRNAs.

### 2.4. Validation by Quantitative Real-Time PCR

Expression analysis of specific miRNAs was validated by quantitative real-time (Q-RT) PCR. cDNA was obtained from 0.5 *μ*g of total RNA using the miScript II reverse transcription kit (Qiagen). Q-RT PCR was performed with QuantiFast SYBR Green PCR Master Mix (Qiagen), using a Rotor Gene Q real-time PCR cycler (Qiagen). Relative expression levels were calculated with the comparative threshold cycle (Cq) method [20], using snRNA U1 as reference for normalization.

### 2.5. Identification of Putative miRNA Targets and Pathway Enrichment Analysis

miRNA target predictions were obtained from TargetScan (http://targetscan.org version 6.2 human). DIANA mirPath v2.0 web-application [21] and overrepresentation analysis (ORA) tool available in ConsensusPathDB (http://cpdb.molgen.mpg.de/CPDB) [22] were utilized in order to identify pathways from Kyoto Encyclopedia of Genes and Genomes (KEGG) database (http://www.genome.jp/kegg/) enriched among the list of mRNA targets regulated by each selected miRNA.

### 2.6. Statistical Analysis

Clinical and Q-RT data were expressed as mean ± SE. Student's *t*-test was used for comparison between the two groups. For correlation analysis, Pearson's coefficient of correlation (*R* ^2^) was calculated, and analysis of variance followed by a Student's *t*-test was used. *P* values ≤ 0.05 were considered significant.

## 3. Results

### 3.1. Characteristics of the Study Population

The characteristics and the main clinical parameters of patients, available in the clinical databank, are summarized in Table 1. LVAD implanted patients were supported for a mean period of 357 days (SE = 66 days). For LVAD implanted patients, mean clinical values measured before the device implant and at the LVAD explant (at the time of heart transplantation) are reported. Although patients in both groups were diagnosed with end-stage heart failure, HTx-LVAD patients showed generally worse baseline clinical parameters compared to HTx-CTRL patients, justifying the requirement for LVAD support due to unstable clinical conditions (Table 1).

**Figure 1:**
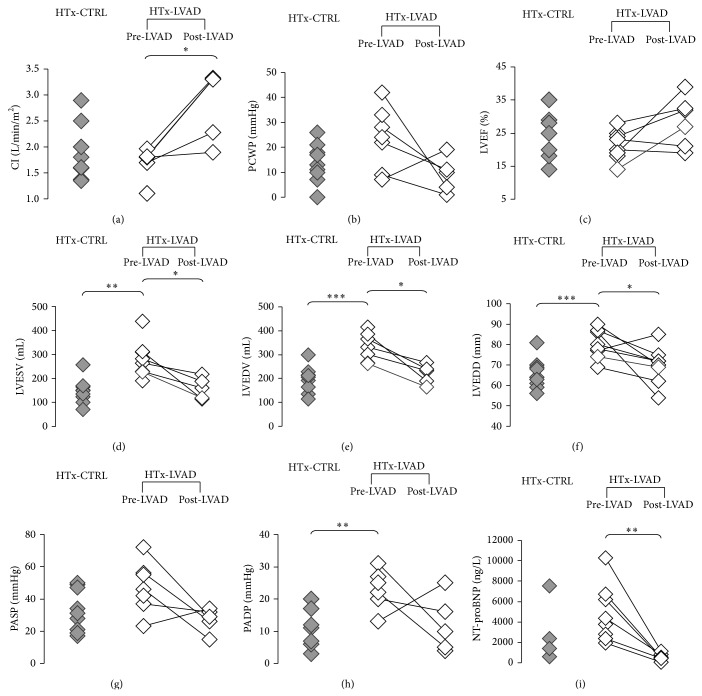
Clinical parameters of individual patients. (a) CI: cardiac index (off-pump); (b) PCWP: pulmonary capillary wedge pressure; (c) LVEF: left ventricular ejection fraction; (d) LVESV: left ventricular end-systolic volume; (e) LVEDV: left ventricular end-diastolic volume; (f) LVEDD: left ventricular end-diastolic diameter; (g) PASP: pulmonary artery systolic pressure; (h) PADP: pulmonary artery diastolic pressure; (i) NT-proBNP: N-terminal prohormone of brain natriuretic peptide. For patients belonging to HTx-LVAD group, available measurements before implantation of assistance device (pre-LVAD) and before heart transplant (post-LVAD) are shown. Statistical significance is calculated according to Student's *t*-test (^*^ *P* < 0.05; ^**^ *P* < 0.01; ^***^ *P* < 0.001). In the HTx-LVAD group, paired *t*-test is used to compare pre-LVAD and post-LVAD values.

Individual data of patients are shown in Figure 1. LVAD support induced a significant increase in the off-pump cardiac index (*P* < 0.05). During LVAD support a decrease in LV end-systolic and end-diastolic volumes and in end-diastolic diameter (*P* < 0.05) has been detected, together with a reduction of the plasmatic levels of NT-proBNP (*P* < 0.01). However, a variable response to mechanical unloading has been observed, with some patients clearly demonstrating an improvement in functional parameters after LVAD support period, while others reporting only a slight change or in some cases even worse data (Figure 1).

### 3.2. miRNA Expression Profiling

In order to get a deeper insight into the molecular mechanisms that are affected by LV mechanical unloading and that could be associated with a potential recovery of heart functionality, miRNA expression profile in LV specimens collected at the moment of heart transplantation from HTx-LVAD and HTx-CTRL patients was compared.

From the heart tissue samples (30–120 mg), an average total RNA concentration of about 400 ng/*μ*L (interquartile range 200 ng/*μ*L) was obtained in RNA eluates. After total RNA extraction, deep sequencing was performed to obtain a complete profile of miRNA expression. Overall, an average of 19 million (range: 7–33 million) clean reads of 50 bp was obtained from each sample sequencing. After preprocessing and filtering steps, 11 million (range: 5–23 million per sample) of reads mapped to mature miRNA sequences annotated in miRBase release 20. On the whole, more than 1700 unique microRNA sequences were detected in heart tissue.

**Figure 2:**
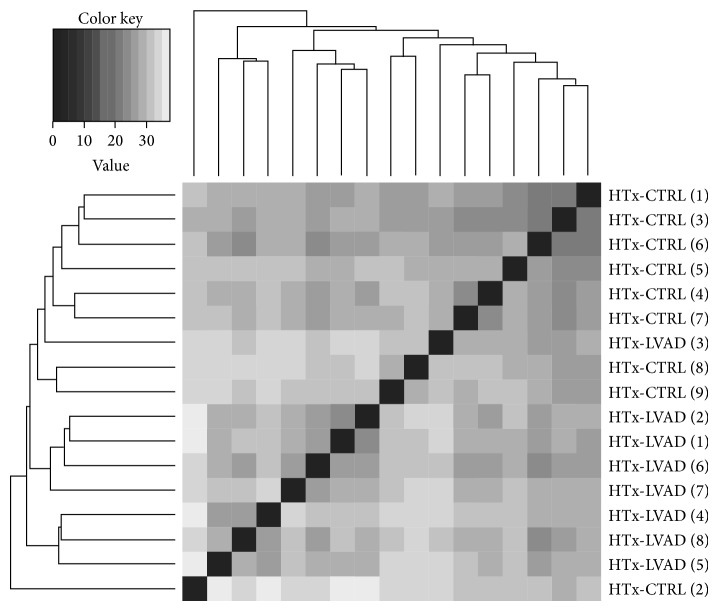
Sample clustering based on sequenced microRNA profiles. Variance-stabilizing transformed count data was used for all samples. The heat map shows a greyscale false colour representation of the Euclidean distance matrix, and the dendrogram represents a hierarchical clustering. Separately, HTx-CTRL and HTx-LVAD samples show a good degree of similarity and group together with the exception of HTx-LVAD (3) and HTx-CTRL (2), which were excluded from downstream analysis.

For each sample, miRNA expression profile was built by counting the number of sequenced reads for each identified miRNA that is present in miRBase v.20 (see Section 2 for more details). In order to retrieve insight into group similarities, sample relations were then investigated by measuring the Euclidean distance between the miRNA expression profiles, followed by hierarchical clustering. Resulting heat map (Figure 2) shows that the miRNA expression profiles well distinguished between HTx-LVAD- and HTx-CTRL samples, despite their wide intrinsic variability. Only samples from 2 patients, HTx-LVAD (3) and HTx-CTRL (2), clustered into inverted group suggesting they could potentially confound our assay. HTx-LVAD (3) patient, who was transplanted in emergency due to sepsis coming from the LVAD driveline, was implanted after a long history of heart failure and did not show any improvement of functional parameters after the period of LVAD support. HTx-CTRL (2) patient, due to a Hodgkin lymphoma 12 years before transplant, received radio and chemotherapy treatments which may have modified molecular responses of the failing heart. For these reasons, these samples have been excluded from downstream analysis.

An analysis of miRNA relative expression levels in the two groups, thus including 8 samples in the HTx-CTRL group and 7 samples in the HTx-LVAD group (after the exclusion of the two outlier patients above specified), was then performed. A total of 23 microRNAs appeared to be differentially expressed (DE), and among these 13 were found upregulated and 10 downregulated, in the HTx-LVAD group with respect to the HTx-CTRL group. The DE miRNAs, starting from those with the highest abundance, are listed in Table 2.

### 3.3. Validation of MicroRNA Expression by Real-Time PCR

**Figure 3:**
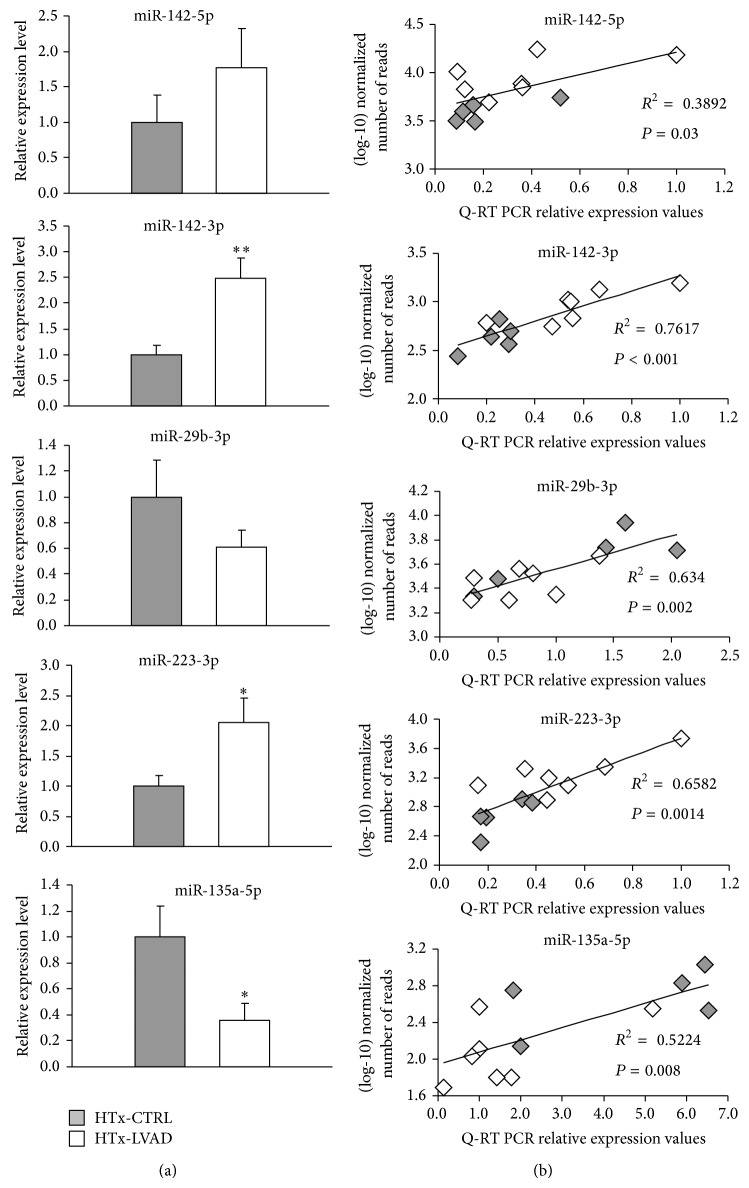
Validation by Q-RT PCR and correlation with next generation sequencing data. (a) Mean Q-RT PCR normalized expression values of selected microRNAs in a subset of HTx-CTRL (*n* = 5, grey bars) and HTx-LVAD (*n* = 7, white bars) heart samples (^*^ *P* < 0.05; ^**^ *P* < 0.01). (b) Correlation analyses between (log10)-transformed number of miRNA read counts and Q-RT PCR relative expression values. Grey and white symbols are representative of HTx-CTRL and HTx-LVAD data pairs, respectively.

Quantitative real-time PCR was performed to validate sequencing results on a small set of DE miRNAs, selected on the basis of the high expression level as well as on the apparent relevance in heart disease according to literature [23–29]. Specifically, miR-142-5p and -3p, miR-29b-3p, miR-223-3p, and miR-135a-5p were chosen for validation test in a subgroup of HTx-CTRL (*n* = 5) and HTx-LVAD (*n* = 7) patients.  Figure 3 shows the relative expression levels as determined by Q-RT experiments for each of the five microRNAs (Figure 3(a)), and the correlation analyses between (log10)-transformed miRNA read counts obtained by NGS and the correspondent relative expression values calculated by Q-RT (Figure 3(b)). Q-RT values appeared to be significantly correlated with sequencing data for all the tested microRNAs (Figure 3(b)), thus sustaining the accuracy and reliability of NGS analysis. For some of the selected miRNAs (miR-142-3p, miR-223-3p, and miR-135a-5p), a significant difference in expression level between HTx-CTRL and HTx-LVAD patients was also confirmed by Q-RT (Figure 3(a)). In other two cases (miR-142-5p and miR-29b-3p), the results indicated a clear consistence with sequencing data, although a statistical significance was not reached probably due to the reduced number of samples used in PCR experiments (Figure 3(a)).

### 3.4. Association between MicroRNA Expression and Clinical Parameters

Besides sequencing data validation, a further analysis has been made in order to check a possible association between the expression of specific microRNAs, identified as differentially regulated in LVAD-supported patients, and their clinical parameters, indicative of heart function.

As shown in Table 1 and Figure 1, there is not a clear distinction in the majority of hemodynamic and echocardiographic parameters measured in patients without and with LVAD support period, because of the worse clinical conditions of HTx-LVAD patients at baseline as well as the interindividual variability in response to LVAD. For these reasons, the association analysis has been limited to the cardiac index, which appeared to be significantly improved in LVAD implanted group with respect to HTx-CTRL patients (2.82 ± 0.30 versus 1.90 ± 0.17 L/min/m^2^, *P* < 0.05) (Table 1).

**Figure 4:**
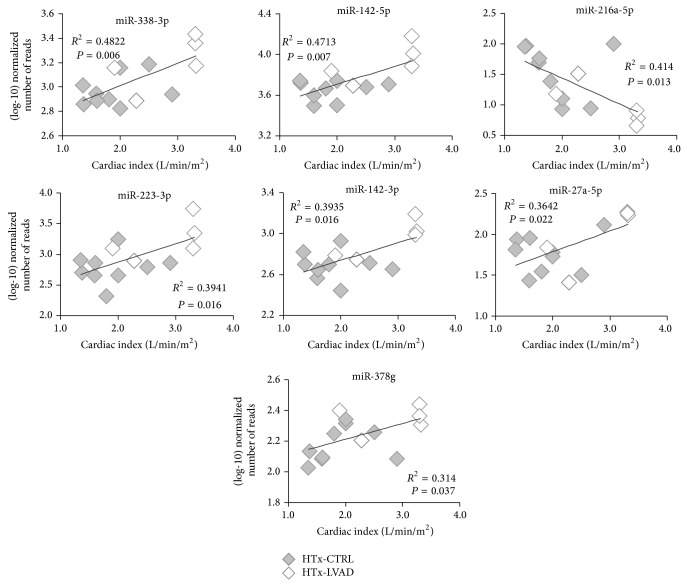
Expression of differentially regulated miRNAs in LVAD-supported patients is associated with cardiac index. The graphs show the correlation analyses between (log10)-transformed number of miRNA read counts and individual measurements of cardiac index in HTx-CTRL (grey symbols, *n* = 9) and HTx-LVAD patients (white symbols, *n* = 5).

As shown in Figure 4, correlation analysis documents a significant association among cardiac index values and expression of some microRNAs (calculated on the basis of number of read counts). In fact, a positive correlation is present with some upregulated miRNAs in HTx-LVAD group: miR-338-3p (*r* = +0.694, *P* = 0.006), miR-142-5p (*r* = +0.686, *P* = 0.007), miR-223-3p (*r* = +0.628, *P* = 0.016), miR-142-3p (*r* = +0.627, *P* = 0.016), miR-27a-5p (*r* = +0.603, *P* = 0.022), and miR-378g (*r* = +0.560, *P* = 0.037). Differently, miR-216a-5p, which is downregulated in LVAD group, appears negatively associated with the cardiac index (*r* = −0.643, *P* = 0.013).

It is important to underline that in the LVAD-supported group the patients who did not demonstrate a recovery in cardiac function and maintained a low cardiac index even after mechanical assistance showed also a correspondence in miRNA expression levels similar to HTx-CTRL patients (Figure 4).

**Figure 5:**
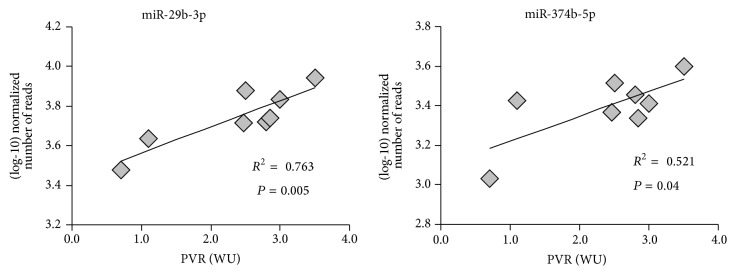
Expression of downregulated miRNAs is associated with pulmonary vascular resistance values in failing transplanted hearts. The graphs show the correlation analyses between (log10)-transformed number of miRNA read counts and individual measurements of pulmonary vascular resistance, expressed in Wood units (WU), in HTx-CTRL patients (*n* = 8).

Moreover, other associations among patients' hemodynamics and miRNA expression were found in relation to pulmonary vascular resistance, calculated as ratio between pulmonary artery pressure and cardiac output values. From clinical database these measurements were available only for patients of the HTx-CTRL group. As shown in Figure 5, a significant positive correlation is present with miR-29b-3p (*r* = +0.873, *P* = 0.005) and miR-374b-5p (*r* = +0.722, *P* = 0.04), two miRNAs which were found highly expressed in heart (Table 2). Both miRNAs appear to be downregulated in LVAD-supported patients compared to the control group (Table 2).

### 3.5. *In Silico* Identification of Putative miRNA Targets and Pathway Enrichment Analysis

The subset of DE microRNAs showing association with cardiac index (miR-338-3p, miR-142-5p and -3p, miR-216a-5p, miR-223-3p, miR-27a-5p, miR-378g) or with pulmonary vascular resistance values (miR-29b-3p and miR-374b-5p) was further analyzed through bioinformatic tools in order to identify putative targets of miRNA regulation and relative pathways enrichment. Prediction of miRNA targets by using DIANA-microT-CDS algorithm and applying a 0.9 cut-off threshold (considered as stringent) [21] revealed a total of 353 putative targets for miR-374b-5p, 350 for miR-29b-3p, 324 for miR-142-5p, 261 for miR-338-3p, 160 for miR-223-3p, 145 for miR-142-3p, 135 for miR-216a, 128 for miR-378g, and 14 for miR-27a-5p. Putative targets of all miRNAs in the subset were then analyzed as a whole to identify the most enriched pathways, as annotated in KEGG database. The complete list of enriched pathways with candidate target genes, ranked according to the number of miRNAs predicted to regulate each pathway and to the significance value calculated by mirPath software, is reported in Supplementary Table 1 in Supplementary Material available online at http://dx.doi.org/10.1155/2014/592512. Among this list, most representative pathways possibly involved in heart function or altered in cardiac disease states are highlighted in Table 3. Remarkably, as shown in the table, several pathways connected with regulation of cellular biomechanics or with different kinds of cardiomyopathies, such as regulation of actin cytoskeleton (with candidate target genes representing 13.5% of total genes annotated in the pathway), focal adhesion (19.3%), dilated cardiomyopathy (16.7%), Wnt signaling pathway (14.4%), hypertrophic cardiomyopathy (16.9%), and ECM (extracellular matrix-) receptor interaction (26.4%), appear among the most regulated pathways by the network of the most relevant miRNAs identified in the study (Table 3). Similar results were also obtained by applying TargetScan context score and evolutionary conservation score algorithms for miRNA target prediction followed by overrepresentation analysis in ConsensusPathDB software (data not shown), to further support the possible involvement of these microRNAs in molecular processes of LVAD-induced heart recovery.

## 4. Discussion

The aim of the study was to exploit a comparison of microRNA modulation in end-stage HF patients who went to transplantation directly or after a period of LVAD support in order to investigate the effects of a mechanical unloading period as well as the potential molecular mechanisms underlying cardiac remodeling. miRNAs can be indeed powerful tools of diagnostic information, either for their extraordinary variability of expression across different physiological and pathological states or for their ability to regulate a large number of target messengers. A next generation sequencing approach has been chosen to have a complete coverage of miRNA profiling and, although the analysis was performed on a limited number of severely ill patients, it reveals significant differences in the patterns of miRNA expression in failing hearts supported or not with LVAD.

The main limit of the study is due to the retrospective nature of the analysis: in fact, only few data, not planned in a specific well defined protocol with timing, are available in the clinical databank, even if there was the authorization of the local ethics committee for tissue samples at the transplantation time. No apex at the time of LVAD implantation was available, in order to allow an internal control analysis. Despite this relevant lack in the materials and methods, the miRNA profiles are capable of distinguishing between the two groups.

Two recent papers have used the NGS approach to compare the miRNA profiling in human heart failure before and after LVAD implantation [10, 12]. The article by Yang et al. [12] is an extensive analysis of the coding and noncoding transcriptome in nonfailing human LV and in failing human LV before/after LVAD support, aimed at identifying an RNA signature that is sensitive to hemodynamic loading conditions as well as capable of discriminating the specific HF etiology. The paper by Akat et al. [10] is a comparative RNA-sequencing analysis of myocardial and circulating miRNAs in patients with stable and end-stage HF before and at different time points after LVAD implantation. Both studies find more than one hundred miRNAs differentially expressed in failing compared to nonfailing hearts, but only a restricted number of them appear to be recovered by LVAD unloading. However, by making a comparison between the specific miRNAs identified in the two studies, low concordance is present: in particular, none of the miRNAs reported as significantly affected by LVAD is common to both screenings. Differently, in our study, miR-27a-5p, significantly upregulated in the HTx-LVAD group of patients, is concordantly found upregulated after LVAD implantation also by Akat et al. [10]. In addition, miR-216a, strongly upregulated both in tissue and in plasma samples of advanced HF patients as reported by Akat et al. [10], showed a more than fivefold decreased expression in HTx-LVAD with respect to HTx-CTRL failing hearts in our findings (Table 2). On the contrary, none of the miRNAs marked as improved or normalized by LVAD in Yang et al. [12] appeared to be significantly changed in our screening, even if in some cases a similar trend in the expression levels in the two groups could be seen (data not shown, not significant difference).

However, it is important to stress that the present study, which utilizes a case-control approach and no paired samples, is focused on the research of miRNAs which are modified by LVAD presence. Since miRNAs are considered important tools for maintaining the cell/tissue/organ homeostasis, their change, considered as “normalization,” might not necessarily be expression of cardiac recovery, but in some cases their “deregulation” might also be signal of a reactive response to a disequilibrium state.

A particularly important finding of this study is that the expression level of some of the identified miRNAs is correlated with the improvement of cardiac index (Figure 4). In the enrolled population, cardiac index (assessed before LVAD implant and off-pump during heart transplant) is the hemodynamic parameter that better differentiates improvement in heart function before and after LVAD support (Table 1). This correlation supports the role of DE miRNAs identified in this screening in cardiac remodeling. In line with this observation, patients who did not recover a cardiac function after LVAD implantation apparently clustered with the HTx-CTRL group (Figure 4). It is worth noting that patients implanted with LVAD had in general worse cardiac parameters compared to the HTx-CTRL group: in fact, an acute phase in patients included in the waiting list for heart transplantation requires an urgent assistance by mechanical support, as suggested by the guidelines, and the worse clinical condition at the LVAD implantation time is a consequence of the disease unstable state. For this reason, although LVAD implantation induced an improvement in cardiac parameters (see Table 1), some values are still worse in patients after LVAD than in HTx-CTRL patients. These considerations suggest that miRNAs modulated by LVAD implantation might indicate the activation of reverse remodeling processes (such as the cytoskeleton-mediated remodeling, see the following) more than representing a marker of the contractility recovery level. In line with our interpretation, 14 miRNAs, out of the 23 selected in the study, were also previously identified as differentially modulated in fetal myocardium compared to nonfailing postnatal hearts [10]. The expression of 10 out of the “fetal” miRNAs appears to be reversed in HTx-LVAD patients, further supporting the hypothesis of reverse remodeling markers. In addition, by considering the positive correlation between the expression levels of miR-29b-3p and miR-374b-5p and the highest pulmonary resistances in the control group (Figure 5), the downregulation of these miRNAs in LVAD patients could play a role in a possible pulmonary resistance decrease after mechanical unloading.

Some of the identified miRNAs have been already described in heart failure or in other cardiovascular diseases. One of the most interesting is miR-142, whose 5p and 3p pre-miRNA strands are both expressed at high level in the performed analysis. Both miR-142-5p and -3p were upregulated about twofold in tissues from LVAD patients, as documented by data reported in Table 2, and their expression was correlated with cardiac index (Figure 4). Altered levels of these miRNAs in blood were previously indicated as potential biomarkers for diagnosis of dilated cardiomyopathy and heart failure [24, 28, 29]. However, this study highlights for the first time that these two miRNAs are modulated in cardiac tissues of LVAD patients. Downregulation of miR-142-5p and -3p has been described in cardiac hypertrophy [30, 31], and a role of these miRNAs as repressors of components of the NF-kB pathway, preventing cytokine-mediated NO production, is suggested [30]. Sarcomeric genes emerged as potential targets of members of the miR-142 family, and modulation of *α*-actinin has been experimentally demonstrated [30]. Indeed, genes of the integrin signaling network connecting membrane and cytoskeleton are also among the pathways identified by genome sequencing as genes associated with functional recovery of end-stage heart failure [32].

A twofold increased expression in LVAD-supported patients was also detected for miR-378 (Table 2), a cardiomyocyte-enriched miRNA which has been reported to act as an endogenous negative regulator of cardiac hypertrophy, and whose levels are downregulated during hypertrophic growth and heart failure [33, 34]. The importance of miR-378 in HF-induced cardiac remodeling is supported by the observation that (a) deficiency of miR-378 alone is sufficient to induce fetal gene expression [34] and (b) compensation of miR-378 loss, through adeno-associated virus (AAV) mediated cardiomyocyte-targeted expression, partially prevents left ventricular hypertrophy and improves cardiac function in an* in vivo *model of cardiac pressure overload by thoracic aortic constriction [33]. These data could be therefore in line with the observed increase in miR- 378 expression in patients showing a positive response to LVAD implantation.

MiR-216a and miR-29b are detected among miRNAs which exhibit a downregulation in cardiac tissues of LVAD patients. MiR-216a was already identified as strongly upregulated in cardiac biopsies from HF patients and positively controlled by TGF-*β* [10, 25]. Concerning miR-29b, the observed downregulation is instead in line with data reported by Matkovich et al. [8] in LVAD-supported heart. The three members of the human miR-29 family have been extensively analyzed in human and animal models for their strong antifibrotic effect in heart and other organs. These miRNAs are indeed predicted to target at least 16 extracellular matrix genes [35]. Other studies have instead indicated that reduction of miR-29 might be cardioprotective by reducing myocardial infarct size and apoptosis in heart subjected to ischemia/reperfusion injury [36]. Moreover, miR-29 has been shown to modulate* de novo *DNA methyltransferase in cardiac tissues, suggesting that a role in epigenetic modifications could contribute to cardiac remodeling [37].

The* in silico *analysis performed to identify putative miRNA targets and the enriched associated pathways reveals several pathways involved in regulation of actin cytoskeleton, focal adhesion, ECM-receptor interaction, and adherens junction (Table 3). The cytoskeleton provides a structural framework for the cell, serving as a scaffold that determines the cell shape and the general organization of the cytoplasm [38]. Embedded within the cytoskeleton, specialized protein structures such as adherens junction and focal adhesion proteins, related to the integrin signaling, facilitate the communication between the cytoskeleton and, respectively, the neighbouring cells or the ECM [39–41]. Recent data clearly demonstrate that cytoskeleton and its related protein complexes regulate many other processes within the cell, including signaling, excitability, impulse propagation, contractility, and gene expression. Therefore, the cytoskeleton acts as a signal integrator for mechanical and structural inputs, and it is supposed to play a pivotal role in myocyte adaptive or maladaptive remodeling processes during physiological growth or pathological conditions. The identification of many genes belonging to these pathways as putative targets of the miRNAs modulated in LVAD patients suggests the involvement of cytoskeleton-mediated remodeling in the recovery process stimulated by LVAD, despite the severe degree of disease in patients at the end stage of heart failure. In line with this hypothesis, a microarray analysis, performed on a small number of patients before and after LVAD implantation, revealed a significant association of integrin signaling pathway with recovery [32].

Moreover, among the most regulated pathways by the subset of miRNAs identified in the presented analysis, PI3K-Akt and mTOR signaling pathways can also be importantly involved in mechanisms controlling the progression of heart failure [42], while the Wnt signaling pathway, normally regulating cardiogenesis during the development, can be induced in adult heart during maladaptive myocardial remodeling [43].

It is also interesting to note that some of the identified miRNAs have been already detected in the plasma of patients with different kind of cardiovascular diseases [10, 23, 27–29]. Although the levels of circulating miRNAs not always reflect the relative level in tissues, it is tempting to hypothesize that these miRNAs have the potential to become novel biomarkers of remodeling processes. A further research is planned in this direction, by considering the potential, future increase of LVAD implantation as partial support and/or temporary mechanical unloading in less severe degree of heart failure, with a possible aim of inducing native heart recovery.

## 5. Conclusions

Despite the limited number of patients together with the retrospective nature of the study, the present miRNA profiling allowed identifying a set of differentially modulated miRNAs in heart tissues of LVAD-supported end-stage heart failure patients. The correlation between the expression level of several of them and hemodynamic functional parameters suggests that dynamic changes in specific miRNAs can be indicative of a recovery process after mechanical unloading. Potential targets of miRNA modulation are implicated in pathways regulating cytoskeleton and interaction with the extracellular matrix, to support the idea that the cytoskeleton, by triggering a wide range of cellular functional responses, might play a pivotal role in guiding the recovery process.

## Supplementary Material

Supplemental Table 1. Putative targets of the nine DE microRNAs showing significant association with cardiac index (miR-338-3p, miR-142-5p and -3p, miR-216a-5p, miR-223-3p, miR-27a-5p, miR-378g) or pulmonary vascular resistance values (miR-29b-3p and miR-374b-5p) were determined according to DIANA-microT-CDS algorithm, by applying a 0.9 cut-off threshold. DIANA mirPath v2.0 application was then employed to perform pathway enrichment analysis by considering the union of all the predicted targets. In the table, the complete list of enriched pathways, as annotated in KEGG database, is ranked on the basis of the number of miRNAs predicted to regulate them and of the significance value calculated by mirPath software.

## Figures and Tables

**Table 1:** Patient characteristics and clinical parameters.

	HTx-CTRL (*n* = 9)	HTx-LVAD (*n* = 8)	
		Pre-LVAD	Post-LVAD
Age (years), mean ± SE	52 ± 3	46 ± 4	—
Male gender (*n*)	5	8	—
Duration of LVAD assistance (days), mean ± SE	—	—	357 ± 66
Ischemic heart disease (IHD) (*n*)	2	1	—

Cardiac index (L/min/m^2^), mean ± SE	1.90 ± 0.17	1.61 ± 0.13	2.82 ± 0.30^#∗∗^
PCWP (mm Hg), mean ± SE	14 ± 3	24 ± 5	9 ± 3^*^
PASP (mm Hg), mean ± SE	33 ± 4	47 ± 6	27 ± 3^*^
PADP (mm Hg), mean ± SE	13 ± 2	23 ± 3^‡^	12 ± 4^*^
LVEF (%), mean ± SE	26 ± 2	21 ± 2	28 ± 3^*^
LVESV (mL), mean ± SE	147 ± 17	282 ± 27^‡^	160 ± 20^**^
LVEDV (mL), mean ± SE	196 ± 18	357 ± 32^§^	219 ± 18^**^
LVEDD (mm), mean ± SE	66 ± 2	80 ± 3^§^	70 ± 3^*^
NT-proBNP (ng/L), mean ± SE	2976 ± 1562	4800 ± 991	576 ± 145^**^

^#^ *P* < 0.05 versus HTx-CTRL; ^‡^ *P* < 0.01 versus HTx-CTRL; ^§^ *P* < 0.001 versus HTx-CTRL; ^*^ *P* < 0.05 versus pre-LVAD; ^**^ *P* < 0.01 versus pre-LVAD.

PCWP: pulmonary capillary wedge pressure; PASP: pulmonary artery systolic pressure; PADP: pulmonary artery diastolic pressure; LVEF: left ventricular ejection fraction; LVESV: left ventricular end-systolic volume; LVEDV: left ventricular end-diastolic volume; LVEDD: left ventricular end-diastolic diameter; NT-proBNP: N-terminal prohormone of brain natriuretic peptide.

**Table 2:** List of differentially expressed (DE) miRNAs in LVAD-supported versus “control” failing explanted hearts.

miRNA	HTx-CTRL	HTx-LVAD	FC	*P* value	*P* adjusted
hsa-miR-29b-3p	5421	3156	0.58	1.11*E* − 03	3.54*E* − 02
**hsa-miR-142-5p**	**4417**	**9146**	**2.07**	2.33**E** − 04	9.28**E** − 03
hsa-miR-4485	3066	329	0.11	6.19*E* − 25	6.41*E* − 22
hsa-miR-374b-5p	2429	1382	0.57	1.16*E* − 03	3.54*E* − 02
hsa-miR-4532	2250	833	0.37	3.91*E* − 07	2.70*E* − 05
hsa-miR-4792	979	412	0.42	2.90*E* − 06	1.76*E* − 04
**hsa-miR-338-3p**	**936**	**1536**	**1.64**	2.25**E** − 03	5.67**E** − 02
**hsa-miR-144-5p**	**873**	**1465**	**1.68**	5.97**E** − 04	2.13**E** − 02
**hsa-miR-223-3p**	**669**	**1947**	**2.91**	2.06**E** − 10	2.67**E** − 08
hsa-miR-135a-5p	593	161	0.27	6.63*E* − 14	1.14*E* − 11
**hsa-miR-142-3p**	**498**	**913**	**1.83**	1.70**E** − 03	4.51**E** − 02
**hsa-miR-335-3p**	**452**	**756**	**1.67**	2.25**E** − 04	9.28**E** − 03
hsa-miR-4284	210	31	0.15	3.28*E* − 19	8.49*E* − 17
hsa-miR-376a-3p	184	75	0.41	1.42*E* − 05	6.66*E* − 04
hsa-miR-3195	159	85	0.53	9.46*E* − 04	3.16*E* − 02
**hsa-miR-378g**	**137**	**271**	**1.98**	2.33**E** − 03	5.69**E** − 02
**hsa-miR-628-5p**	**109**	**193**	**1.77**	1.32**E** − 03	3.91**E** − 02
**hsa-miR-5683**	**98**	**415**	**4.23**	1.26**E** − 19	4.36**E** − 17
**hsa-miR-146b-3p**	**88**	**251**	**2.85**	4.95**E** − 06	2.85**E** − 04
**hsa-miR-23a-5p**	**67**	**119**	**1.77**	2.96**E** − 03	6.64**E** − 02
**hsa-miR-27a-5p**	**61**	**126**	**2.05**	9.06**E** − 05	3.91**E** − 03
**hsa-miR-4461**	**58**	**118**	**2.04**	2.42**E** − 03	5.69**E** − 02
hsa-miR-216a-5p	50	8	0.17	5.30*E* − 13	7.84*E* − 11

Differentially expressed miRNAs are ranked according to the number of read counts. Upregulated and downregulated miRNAs are, respectively, shown in bold and white lines. FC = fold change.

**Table 3:** Representative enriched KEGG pathways, involved in heart function or disease, according to *in silico* analysis of predicted targets of miRNAs associated with clinical parameters in failing heart with/without LVAD support.

KEGG pathway	*P* value	Number of miRNAs	Number of target genes (% in the pathway)
Regulation of actin cytoskeleton	1.44*E* − 04	9	29 (13.5%)
Focal adhesion	1.37*E* − 16	8	40 (19.3%)
PI3K-Akt signaling pathway	1.15*E* − 11	8	50 (14.4%)
mTOR signaling pathway	9.89*E* − 06	8	13 (21.7%)
Dilated cardiomyopathy	5.46*E* − 05	8	15 (16.7%)
Wnt signaling pathway	6.04*E* − 05	8	20 (14.4%)
Hypertrophic cardiomyopathy (HCM)	7.65*E* − 05	8	14 (16.9%)
Arrhythmogenic right ventricular cardiomyopathy	1.77*E* − 03	8	13 (17.6%)
ECM-receptor interaction	3.21*E* − 53	7	23 (26.4%)
MAPK signaling pathway	1.39*E* − 06	7	36 (14.0%)
Cytokine-cytokine receptor interaction	5.73*E* − 03	7	24 (9.1%)
TGF-beta signaling pathway	9.21*E* − 06	6	16 (20.0%)
Adherens junction	4.53*E* − 04	6	11 (15.1%)
Phosphatidylinositol signaling system	3.51*E* − 03	5	12 (14.8%)
