# Supplementary material for: Differential Regulation of MicroRNAs in End-Stage Failing Hearts Is Associated with Left Ventricular Assist Device Unloading
Source: Biomed Res Int. 2015 Feb 1;2015:592512. doi: 10.1155/2015/592512 (PMC4330954; doi:10.1155/2015/592512)
Supplement: Supplementary file 1 — Supplemental Table 1. Putative targets of the nine DE microRNAs showing significant association with cardiac index (miR-338-3p, miR-142-5p and -3p, miR-216a-5p, miR-223-3p, miR-27a-5p, miR-378g) or pulmonary vascular resistance values (miR-29b-3p and miR-374b-5p) were determined according to DIANA-microT-CDS algorithm, by applying a 0.9 cut-off threshold. DIANA mirPath v2.0 application was then employed to perform pathway enrichment analysis by considering the union of all the predicted targets. In the table, the complete list of enriched pathways, as annotated in KEGG database, is ranked on the basis of the number of miRNAs predicted to regulate them and of the significance value calculated by mirPath software. [file 592512.f1.pdf]

Supplemental Material

Supplemental Table 1. List of the most enriched KEGG pathways according to *in silico* analysis of predicted targets of miRNAs associated with clinical parameters in failing heart with/without LVAD support.

KEGG pathway	p value	n° miRNAs	n° target genes (% in the pathway)	candidate target genes
Pathways in cancer	2.95E-09	9	49 (15.0%)	FZD7; WNT16; FZD5; TGFBR1; APC; CRK; WNT5A; HHIP; COL4A5; PIK3R5; ETS1; RALA; WNT3; IGF1R; FGF20; WNT5B; CDK6; MST1; EPAS1; MITF; PTK2; CBLB; ITGAV; CDH1; FZD10; PDGFB; KIT; VEGFC; COL4A3; PIK3R1; COL4A4; FGF9; KITLG; RAC1; FGF18; BMP2; MAX; LAMC1; IGF1; CASP8; LEF1; COL4A6; LAMA2; STAT1; PTEN; FOXO1; TGFBR2; TFG; COL4A1
Regulation of actin cytoskeleton	1.44E-04	9	29 (13.5%)	WASL; ITGB8; APC; CRK; MYL12B; PIK3R5; ROCK2; MYH10; GNG12; TMSB4Y; MYLK2; FGF20; ITGA1; RRAS2; PTK2; ITGAV; PIKFYVE; NCKAP1L; PDGFB; CFL2; PIK3R1; FGF9; RAC1; FGF18; MYL7; PDGFC; MYLK3; NCKAP1; DIAPH2
Focal adhesion	1.37E-16	8	40 (19.3%)	ITGB8; CRK; THBS1; MYL12B; COL4A5; PIK3R5; RAP1A; COL27A1; ROCK2; IGF1R; MYLK2; CAV2; ITGA1; COL3A1; PTK2; ITGAV; COL2A1; COL5A1; PDGFB; COL1A1; VEGFC; COL4A3; PIK3R1;

				COL4A4; RAC1; MYL7; COL1A2; LAMC1; IGF1; PDGFC; MYLK3; COL11A1; COL6A3; COL4A6; LAMA2; COL5A3; PTEN; COL5A2; ARHGAP5; COL4A1
PI3K-Akt signaling pathway	1.15E-11	8	50 (14.4%)	PRLR; ITGB8; THBS1; CREB5; COL4A5; PIK3R5; COL27A1; GNG12; STK11; IGF1R; FGF20; PPP2R5C; ITGA1; CDK6; COL3A1; IL7R; EFNA5; GHR; PTK2; ITGAV; COL2A1; TSC2; JAK2; EIF4E; COL5A1; PDGFB; COL1A1; KIT; VEGFC; COL4A3; PIK3R1; COL4A4; FGF9; KITLG; RAC1; FGF18; COL1A2; LAMC1; IGF1; PDGFC; COL11A1; COL6A3; COL4A6; FOXO3; LAMA2; COL5A3; PTEN; COL5A2; RPS6KB1; COL4A1
Transcriptional misregulation in cancer	4.27E-07	8	30 (16.8%)	CCNT2; WNT16; LMO2; ELK4; HMGA2; AFF1; MLLT3; IGF1R; PRCC; UTY; PTK2; ATM; CEBPB; MLF1; NR4A3; KLF3; BMP2K; PDGFB; JMJD1C; ETV1; CDK14; MAX; DOT1L; IGF1; MYCN; MEF2C; SIX4; FOXO1; SMAD1; TGFBR2
mTOR signaling pathway	9.89E-06	8	13 (21.7%)	PIK3R5; STK11; RPS6KA1; RICTOR; AKT1S1; TSC2; EIF4E; VEGFC; PIK3R1; IGF1; PTEN; ULK2; RPS6KB1
Axon guidance	4.22E-05	8	20 (15.7%)	SEMA5A; EPHA5; ROCK2; NTN1; SEMA4F; EFNA5; NCK1; PTK2; RASA1; UNC5A; NFAT5; SRGAP3; NCK2; DPYSL5; CFL2;

				SEMA3D; RAC1; ROBO1; SEMA6D; GNAI1
Dilated cardiomyopathy	5.46E-05	8	15 (16.7%)	DES; ITGB8; TPM1; ADCY2; CACNB4; SGCD; ITGA1; ITGAV; DMD; PLN; SLC8A1; IGF1; LAMA2; CACNA2D1; ATP2A2
Wnt signaling pathway	6.04E-05	8	20 (14.4%)	FZD7; WNT16; FZD5; LRP6; TBL1X; BTRC; APC; WNT5A; PORCN; ROCK2; WNT3; PPP2R5C; WNT5B; CAMK2A; NFAT5; GPC4; FZD10; RAC1; SIAH1; LEF1
Hypertrophic cardiomyopathy (HCM)	7.65E-05	8	14 (16.9%)	DES; ITGB8; TPM1; CACNB4; SGCD; ITGA1; ITGAV; DMD; SLC8A1; PRKAB2; IGF1; LAMA2; CACNA2D1; ATP2A2
Arrhythmogenic right ventricular cardiomyopathy	1.77E-03	8	13 (17.6%)	DES; ITGB8; CACNB4; SGCD; ITGA1; ITGAV; DMD; SLC8A1; DSG2; LEF1; LAMA2; CACNA2D1; ATP2A2
Endocytosis	7.59E-03	8	23 (11.3%)	TGFBR1; SMAD6; STAM; IQSEC3; WWP1; PDCD6IP; AP2B1; SMURF2; CHMP1B; IGF1R; CAV2; CBLB; PSD3; RAB11FIP2; RAB11A; KIT; RAB11FIP5; HGS; HSPA8; VPS37C; TGFBR2; PIP5KL1; ASAP2
Leukocyte transendothelial migration	1.05E-02	8	15 (12.7%)	CTNND1; CLDN8; MYL12B; PIK3R5; RAP1A; ROCK2; CLDN16; PTK2; PIK3R1; RAC1; MYL7; CLDN1; CDH5; ARHGAP5; GNAI1
Neurotrophin signaling pathway	2.10E-02	8	15 (12.5%)	NTF3; CAMK4; CALM3; CRK; PIK3R5; RAP1A; NTRK3; RPS6KA1; CAMK2A; KIDINS220; PIK3R1; RAC1; GAB1; FOXO3; IRAK1

Salivary secretion	3.61E-02	8	11 (12.2%)	ADCY2; CALM3; ATP1B1; ATP2B1; ATP1B3; RYR3; GNAQ; GUCY1A2; PRKG1; ATP1A1; ITPR2
ECM-receptor interaction	3.21E-53	7	23 (26.4%)	ITGB8; SV2B; THBS1; COL4A5; COL27A1; ITGA1; COL3A1; ITGAV; COL2A1; COL5A1; COL1A1; COL4A3; COL4A4; COL1A2; LAMC1; COL11A1; COL6A3; COL4A6; LAMA2; COL5A3; COL5A2; CD47; COL4A1
MAPK signaling pathway	1.39E-06	7	36 (14.0%)	NTF3; CACNA1A; TGFBR1; RASA2; ELK4; CRK; CACNB4; RAP1A; GNG12; MAP4K3; MAP4K4; RPS6KA1; FGF20; TAB2; MAP3K11; RRAS2; MST1; TAOK1; CACNA1I; DUSP10; RASA1; MAP3K12; RASGRP1;PPM1A; PDGFB; FGF9; RAC1; FGF18; HSPA8; NF1; MAX; TNFRSF1A; MAP3K2; CACNA2D1; MEF2C; TGFBR2
Melanoma	1.48E-04	7	13 (18.3%)	PIK3R5; IGF1R; FGF20; CDK6; MITF; CDH1; PDGFB; PIK3R1; FGF9; FGF18; IGF1; PDGFC; PTEN
Pancreatic secretion	3.25E-03	7	13 (13.5%)	ADCY2; TRPC1; RAP1A; SLC4A4; ATP1B1; ATP2B1; ATP1B3; RAB11A; RAC1; GNAQ; ATP2A2; ATP1A1; ITPR2
mRNA surveillance pathway	5.01E-03	7	12 (13.2%)	MSI2; CPSF7; PAPOLG; PABPC4; PPP2R5C; NXF1; PABPC1L; PABPC4L; SMG1; FIP1L1; ACIN1; GSPT1
Cytokine-cytokine receptor interaction	5.73E-03	7	24 (9.1%)	PRLR; TGFBR1; INHBB; CXCL5; TNFRSF1B; IL7R; CCL11; GHR; CCL7; LIFR; PDGFB;

				KIT; VEGFC; ACVR2A; KITLG; BMP2; PDGFC; BMPR1A; TNFRSF1A; IL6ST; IL11RA; TNFRSF9; TGFBR2; BMPR2
Gastric acid secretion	7.52E-03	7	11 (14.7%)	ADCY2; CALM3; ATP1B1; MYLK2; CAMK2A; ATP1B3; GNAQ; MYLK3; ATP1A1; ITPR2; GNAI1
Bile secretion	7.52E-03	7	10 (13.9%)	ADCY2; AQP9; SLC4A4; ATP1B1; AQP4; ATP1B3; ABCC3; SLC22A7; ATP1A1; SCARB1
ErbB signaling pathway	1.19E-02	7	13 (14.9%)	HBEGF; NRG4; CRK; PIK3R5; CAMK2A; NCK1; PTK2; CBLB; NCK2; PIK3R1; GAB1; ABL2; RPS6KB1
Ubiquitin mediated proteolysis	1.25E-02	7	17 (12.4%)	BTRC; FBXW7; TRIM37; UBR5; WWP1; SMURF2; UBE4A; RHOBTB1; HERC3; CBLB; CUL5; UBE2A; UBE2Q1; SIAH1; ERCC8; KLHL9; UBE2W
Bacterial invasion of epithelial cells	1.41E-02	7	10 (13.2%)	WASL; CRK; PIK3R5; CAV2; PTK2; CBLB; CDH1; PIK3R1; RAC1; GAB1
Viral myocarditis	4.73E-02	7	9 (15%)	CXADR; MYH10; SGCD; DMD; RAC1; MYH15; CASP8; LAMA2; ABL2
Protein digestion and absorption	1.37E-16	6	24 (27.0%)	COL4A5; ELN; COL27A1; COL7A1; ATP1B1; COL3A1; COL9A1; COL2A1; COL15A1; ATP1B3; SLC7A7; COL5A1; COL1A1; SLC8A1; COL4A3; COL4A4; COL1A2; COL11A1; COL6A3; COL4A6; ATP1A1; COL5A3; COL5A2; COL4A1
TGF-beta signaling	9.21E-06	6	16 (20.0%)	TGFBR1; SMAD6; INHBB; THBS1; SMURF2;

pathway				ROCK2; DCN; ACVR2A; BMP2; TFDP1; BMPR1A; LTBP1; SMAD1; TGFBR2; BMPR2; RPS6KB1
Small cell lung cancer	2.65E-05	6	15 (17.4%)	COL4A5; PIK3R5; CDK6; APAF1; PTK2; ITGAV; COL4A3; PIK3R1; COL4A4; MAX; LAMC1; COL4A6; LAMA2; PTEN; COL4A1
Melanogenesis	2.15E-04	6	16 (15.8%)	FZD7; WNT16; FZD5; ADCY2; CALM3; WNT5A; WNT3; WNT5B; MITF; CAMK2A; FZD10; KIT; KITLG; GNAQ; LEF1; GNAI1
Adherens junction	4.53E-04	6	11 (15.1%)	TGFBR1; WASL; CTNND1; SORBS1; IGF1R; CDH1; FER; RAC1; LEF1; PVRL3; TGFBR2
Lysine degradation	3.25E-03	6	7 (13.7%)	SETDB2; SETD2; NSD1; ASH1L; DOT1L; SUV420H2; MLL3
Endocrine and other factor-regulated calcium reabsorption	9.63E-03	6	8 (16.7%)	AP2B1; ATP1B1; ATP2B1; ATP1B3; RAB11A; SLC8A1; GNAQ; ATP1A1
RNA degradation	2.28E-02	6	10 (13.5%)	CNOT6; CNOT4; SKIV2L2; PABPC4; C1D; PAN2; PABPC1L; PABPC4L; CNOT3; DHX36
Amoebiasis	8.37E-14	5	21 (19.3%)	COL4A5; PIK3R5; COL27A1; SERPINB9; COL3A1; PTK2; COL2A1; COL5A1; COL1A1; COL4A3; PIK3R1; COL4A4; COL1A2; LAMC1; GNAQ; COL11A1; COL4A6; LAMA2; COL5A3; COL5A2; COL4A1
Basal cell carcinoma	1.25E-04	5	11 (20.0%)	FZD7; WNT16; FZD5; APC; WNT5A; HHIP; WNT3; WNT5B; FZD10; BMP2; LEF1

p53 signaling pathway	2.03E-04	5	12 (17.6%)	THBS1; CDK6; APAF1; ATM; TSC2; SIAH1; MDM4; IGF1; CASP8; PTEN; CCNG2; PPM1D
Phosphatidylinositol signaling system	3.51E-03	5	12 (14.8%)	CALM3; PIK3R5; PIKFYVE; INPP4A; PIK3R1; DGKB; OCRL; INPP5A; DGKD; PTEN; ITPR2; DGKH
Hedgehog signaling pathway	1.13E-02	5	8 (15.7%)	WNT16; BTRC; WNT5A; HHIP; WNT3; RAB23; WNT5B; BMP2
Aldosterone-regulated sodium reabsorption	3.47E-02	5	6 (15.4%)	PIK3R5; ATP1B1; ATP1B3; PIK3R1; IGF1; ATP1A1
Renal cell carcinoma	3.54E-02	5	10 (15.2%)	CRK; PIK3R5; RAP1A; ETS1; EPAS1; PDGFB; VEGFC; PIK3R1; RAC1; GAB1
Pancreatic cancer	4.73E-02	5	9 (13.6%)	TGFBR1; PIK3R5; RALA; CDK6; VEGFC; PIK3R1; RAC1; STAT1; TGFBR2
Mineral absorption	4.22E-05	4	11 (21.6%)	ATP7A; ATP1B1; ATP2B1; ATP1B3; STEAP2; SLC11A2; SLC8A1; ATP1A1; CYBRD1; SLC46A1; SLC30A1
